# Skin temperature reveals the intensity of acute stress

**DOI:** 10.1016/j.physbeh.2015.09.032

**Published:** 2015-12-01

**Authors:** Katherine A. Herborn, James L. Graves, Paul Jerem, Neil P. Evans, Ruedi Nager, Dominic J. McCafferty, Dorothy E.F. McKeegan

**Affiliations:** Institute of Biodiversity, Animal Health and Comparative Medicine College of Medical, Veterinary & Life Sciences, University of Glasgow, Glasgow, UK

**Keywords:** Stress-induced hyperthermia, Animal welfare, Thermal imaging, Corticosterone

## Abstract

Acute stress triggers peripheral vasoconstriction, causing a rapid, short-term drop in skin temperature in homeotherms. We tested, for the first time, whether this response has the potential to quantify stress, by exhibiting proportionality with stressor intensity. We used established behavioural and hormonal markers: activity level and corticosterone level, to validate a mild and more severe form of an acute restraint stressor in hens (*Gallus gallus domesticus*). We then used infrared thermography (IRT) to non-invasively collect continuous temperature measurements following exposure to these two intensities of acute handling stress. In the comb and wattle, two skin regions with a known thermoregulatory role, stressor intensity predicted the extent of initial skin cooling, and also the occurrence of a more delayed skin warming, providing two opportunities to quantify stress. With the present, cost-effective availability of IRT technology, this non-invasive and continuous method of stress assessment in unrestrained animals has the potential to become common practice in pure and applied research.

## Highlights

• We measured skin temperature in hens following a mild or more severe acute stressor.
• The temperature of thermoregulatory tissues temporarily dropped under acute stress.
• The magnitude of this skin temperature change reflected acute stressor intensity.
• Infrared thermography offers a non-invasive method of stress assessment.

## 1 Introduction

Stress is a complex, multidimensional phenomenon of great biological importance, but challenging to assess [1]. Under acute stress, sympathetically-mediated vasoconstriction causes a rapid drop in skin temperature, and this influx of peripheral blood, along with stress-induced thermogenesis, simultaneously increases core temperature [2], [3]. As with established hormonal stress markers, the core temperature increase, termed ‘stress-induced hyperthermia’ (SIH), is proportional to stressor intensity [4], and forms the basis of new anxiety assays in pharmaceutical research [4] and animal welfare assessment [5]. However, as with blood sample collection for hormone assays [6], the act of inserting or implanting a probe to measure core temperature is invasive and, if applied within the period of measurement, is in itself a stressor [7]. Indeed, capture and handling can elicit an acute stress response [8], making the assessment of stress in wild or free-ranging, non-instrumented animals particularly challenging [9]. Skin temperature, in contrast, can be measured non-invasively using infrared thermography (IRT) [10]. If similarly proportional to stressor intensity, measuring stress via the drop in skin temperature rather than using established, invasive methods has benefits with regards to animal welfare. In addition, this approach would allow continuous collection of data throughout the stress response, without the confounding effects of repeated capture and re-sampling [10].

**Fig. 1:**
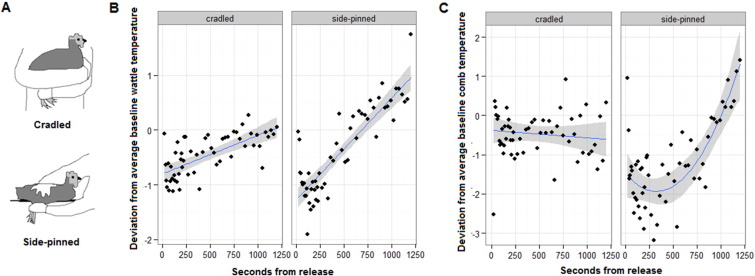
Handling effects on skin temperature. (A) Illustrates a mild and more stressful hold: cradling and side-pinning. Plots show post-handling temperature deviation ± S.E. from individuals' own baseline temperature (0) for the wattle (B) and comb (C), with the mean per instantaneous sampling interval shown (points).

Skin temperature measurement by IRT requires bare skin [10]. Different regions of the skin vary in whether exhibit temperature changes under acute stress, for example in humans, cooling occurs on the nose but not the cheeks [11]. The naturally bare face, comb and wattle, in addition to the eye, make the chicken an excellent model for comparing amongst potential skin regions in the development of this method. In chickens, gentle ‘cradle’ handling (Fig. 1A) is stressful: previous work has shown that catecholamine levels rise immediately [8], and comb and eye temperature drop within a minute by around 2 °C and 0.8 °C, respectively [12], whilst core temperature increases around 0.5 °C over 9–12 min [13]. This is distinct from thermal changes under heat stress, where catecholamine and corticosterone levels also increase [14] but facial- and core temperature are positively correlated [15]. More physically restrictive forms of handling than cradling elicit a proportionately stronger acute hormonal stress response [16]. We examined behavioural activity level and corticosterone levels in the 20 min following exposure to cradling or a more restrictive hold: side-pinning (Fig. 1A), to demonstrate that these constitute a mild and more stressful handling technique respectively. Accordingly, we measured skin temperature using IRT after applying these handling techniques to test whether skin temperature changes differed between the two levels of acute stressor intensity.

## 2 Materials and methods

### 2.1 Husbandry and ethical statement

Trials were conducted from March–June 2013 at Cochno Farm & Research Centre, near Glasgow. One hundred female, 16-week old, non-beak trimmed Lohmann Brown pullets were obtained from a commercial supplier, with industry standard vaccinations prior to arrival. On arrival, hens were fitted with a unique leg ring and housed in 10 groups of 10 in adjacent 1 m × 2 m pens. Pens were equipped with litter of wood shavings, a nest box that provided a perch, and pecking objects (CDs arranged on strings) and daily scattered seed for further enrichment. Layers mash and water were available ad libitum. We waited to start trials until 19 weeks, when all hens were confirmed to be laying, in case hormonal state or changed energy investment altered body temperature. From 19 weeks, one group per week was caught and moved by crate within 5 min to an L-shaped arena (4 m^2^; 3 m^2^ rectangular ‘home pen’ attached by side-door to 1 m^2^ square ‘video area’) in another barn, where the same husbandry was applied. Between arrival at the farm and testing, hens were handled only for ringing and for transportation to the L-shaped arena. Room temperature was maintained within the thermal neutral zone at 18°C (range 18.0–18.3 during filming) and a 14 h:10 h light:dark cycle.

Hens were attended by an on-call veterinarian who routinely inspected feather condition and health every 1–2 weeks. We aimed to score feather loss, in case this altered stress levels or thermoregulation, however none occurred. Five hens sustaining a superficial injury were immediately identified, treated, and excluded from the study. After trials, 74 hens were re-homed by local hobbyists and 26 were retained by the University of Glasgow for further study. Research was conducted under Home Office license (60/4466), and subject to ethical review at the University of Glasgow.

### 2.2 Habituation to the experimental apparatus

Each group spent one week in the L-shaped arena. Outside of trials, the side-door was left open to allow hens free access to the entire pen. Trials were staggered over days 5–7. On each of the 2 days prior to a hen's own test day, we waited until the focal hen entered the video area voluntarily and trapped her there for 20 min. The aim of these ‘isolation’ trials was to habituate hens to the short-term physical (but not visual or auditory) isolation from the group necessary for unobstructed filming on the test day. To evaluate the effect of isolation in the film area on skin temperature, comb, eye, wattle and face temperature collected at 100 ± 10s intervals were compared across the two isolation trials and also a 20 min isolation on the test day (the ‘unhandled’ trial, Section 2.3, see Section 2.4 for temperature measurement methods). A gradual 1.2°C increase in wattle temperature and 2.6°C decrease in comb temperature during isolation trial 1 had flattened by the unhandled trial (removal of day x seconds into isolation: wattle likelihood ratio test with chi-squared distribution, LRT X^2^_2_ = 11.19, p = 0.004, comb LRT X^2^_2_ = 10.98, p = 0.004). The reduction in temporal patterns suggests that repetition of the protocol overcame some initial isolation stress before the test day. Eye temperature showed a small, linear decrease of around 0.2°C over the 20 min isolations (removal of seconds into isolation main effect LRT X^2^_1_ = 6.55, p = 0.01), and this did not change over days (prior removal of day × seconds into isolation interaction LRT X^2^_2_ = 4.32, p = 0.12). However, eye temperature was around 0.4°C cooler during the two isolation trials than the unhandled trial (removal of day main effect LRT X^2^_2_ = 102.32, p < 0.0001), again suggesting a reduction in isolation stress by the test day [10]. Face temperature showed no temporal patterns within or across day (removal of day x seconds into isolation interaction LRT X^2^_2_ = 4.32, p = 0.12; main effect of day LRT X^2^_2_ = 1.10, p = 0.58, main effect of seconds into isolation LRT X^2^_1_ = 3.30, p = 0.07). Data were analysed with models specifying the same covariates and random effects as our main analyses (described in Section 2.6).

### 2.3 Handling trial

On the test day, we videoed the focal hen with a thermal imaging camera (FLIR SC640™, 15 FPS, sensitivity < 0.1 °C, accuracy ± 2%), once during 20 min of isolation when she had voluntarily entered the video area and was unhandled, as during isolation training, and once during 20 min of isolation following capture and 30s of either cradling or side-pinning (see Fig. 1). We examined skin temperature for 20 min on the expectation that corticosterone levels and hence the stress response would peak within this time [17]. Per group, we tested 6–7 hens, giving a final sample size of 57 hens, 30 of which were randomly assigned to the cradled treatment and 27 side-pinned. ‘Unhandled’ and ‘handled’ trials were spaced 120–150 min apart, with the order randomized across hens. After trials, hens were weighed and photographed from the side. Photo pixel count was converted to comb and wattle area relative to a within-image scale, using ImageJ™. Body weight (F_1,55_ = 0.03, p = 0.87), comb size (F_1,55_ = 0.38, p = 0.54) and wattle size (F_1,55_ = 2.88, p = 0.1) did not differ between hens of the two handling treatments, and were included as covariates in analyses (see Section 2.6).

### 2.4 Data extraction

Still images were selected from the video using FLIR ResearchIR™. From images, we used a drawing tool in FLIR Thermocam Researcher Pro 2.10™ to delineate the comb, wattle and face (head excluding comb and wattle) and extract the maximum temperature of these along with the temperature at the centre of the eye. Emissivity was set to 0.97. Subjectivity in delineating facial regions was minimal, with within and between observer repeatabilities of temperatures for each facial region > 95%. Comparing intervals of 10s-1min, we opted to extract a still image every 10 s ± 4 s for 0–4 min and 30 s ± 10 s thereafter for the handling and unhandled trials, with gaps in the time series where no suitable image was available. Per image, body position descriptors noted were: ‘head position’ (above/below shoulders), ‘face angle’ (chin up/flat/down), ‘head tilt’ (angled toward the cage front/side on/back) and ‘side’ (left/right of face). Behaviour in the 5 s preceding each image was categorized as active or inactive. A blackbody trial identified a 0.5 °C/m drop in temperature recorded by the camera, including an effect of filming through caging, so distance was categorized (front/middle/rear third of video area).

### 2.5 Hormonal and behavioural validation of the handling stressors

To assess whether cradling and side-pinning successfully induced a mild and more severe state of acute stress respectively, we validated the cradled and side-pinning hold using established hormonal and behavioural markers.

For the hormonal response, we examined corticosterone at 20 min following handling. Five hens per group from the main study were retained after the main trial, of which 21 (2–3 per group) repeated the trial. Consecutive groups spent 4 days in the L-shaped pen, with days 1 and 2 undisturbed for settling, day 3 for the baseline phase, and day 4 the handling phase. For the baseline phase, the focal hen was isolated in the video area, as during the unhandled trial, and captured at 20 min for blood-sampling. In the handling phase, hens were held for 30 s, released into the video area, and then recaptured for blood-sampling at 20 min post-first capture. Hens were blood sampled from the brachial vein, with ≥ 1 h between individuals and sampling within 2 min of capture, to avoid group disturbance or individual re-capture effects on corticosterone levels respectively [6]. Corticosterone concentrations were determined in plasma samples following a standard diethyl ether extraction (50 μl plasma, 5 ml diethyl ether, vortex, centrifuge, decant solvent using a methanol dry ice bath, dry and reconstitute in 600 μl calibrator diluent (from ELISA)) using a commercial ELISA (Caymen Chemical Company, Ann Arbor, MI, USA) used according to the manufacturer's instructions. Four hens were excluded due to failure to sample within 3 min and/or unreliable assays (poor sample quality or coefficient of variation > 10% across triplicate) in one or both phases, giving a sample size of 17 hens (n = 10 cradled, 7 side-pinned). Side-pinning hens for 30 s resulted in a greater elevation of plasma corticosterone, from individuals' own baseline level, than cradling hens for the same duration (mean ± s.d. baseline 2.02 ± 0.91 ng/ml, cradled 2.57 ± 1.51 ng/ml, side-pinned 3.21 ± 1.48 ng/ml; removing handling method LRT X^2^_2_ = 7.1, p = 0.029, ID nested in group were random effects). Weight and time of day were covariates in the model, with weight positively correlated with corticosterone level (LRT X^2^_1_ = 5.71, p = 0.017) and no significant effect of time of day (LRT X^2^_1_ = 0.97, p = 0.33).

With regard to behavioural validation, reduced activity level is a known behavioural marker of stress in poultry [18]. To examine the behavioural response to handling in the main trial, therefore, we used the behavioural categorizations (active or inactive) collected at approximately 30s ± 10 s intervals (with still images) throughout the main trials as point samples of activity. We used logistic regression to compare these 30s instantaneous behavioural scans (two-level factor, 0 inactive, 1 active) between unhandled, cradled and side-pinned trials, with time of day and weight covariates and ID nested in group a random effect to account for repeated measures. Activity level was lower in the 20 min post-handling with increasing handling stressfulness (% of instantaneous behavioural scans at 30 s intervals: unhandled 94%, post-cradling 84% and post-side-pinning 70%; removing trial from model: LRT X^2^_2_ = 217.24, p < 0.0001). Weight (LRT X^2^_1_ = 0.01, p = 0.93) and time of day (LRT X^2^_1_ = 0.06, p = 0.82) were non-significant.

Together, these hormonal and behavioural data validate cradling and side-pinning as a mild and more severe manipulation of acute stress.

### 2.6 Statistical methods

Data were analysed with R version 3.1.3 (R Core Team, 2014, http://www.R-project.org/).

The skin temperature of each facial region was analysed separately. Over the two isolation training sessions and the unhandled trial, we observed highly repeatable differences in temperature amongst hens not subject to handling (ANOVA wattle: r = 0.62; comb: r = 0.58; eye: r = 0.60; face: r = 0.55, all n = 57, all p < 0.001). Accordingly, temperature values were expressed as deviations from individuals' own average temperature in the corresponding skin region during the unhandled trial, termed their ‘baseline temperature’. Baseline temperature for the corresponding skin region was also included as a covariate in each model, in case individuals with higher baseline temperature had greater scope to cool under stress, and vice versa. Bird identities within group were random effects, to control for repeated measures. If handling induced an acute stress response, we would expect skin temperature to drop with vasoconstriction and possibly increase above baseline levels with subsequent heat dissipation [12], [13], so we tested for linear and quadratic temporal patterns. To test for a difference in the temporal pattern in skin temperature between the two stressor intensities, we specified interactions between seconds from release from the hold (quadratic then linear expression) and trial type (unhandled, cradled or side-pinned). The significance of these interactions was tested using LRT between models with and without these variables. Baseline temperature, time of day, distance from the camera, descriptors of body position, order of phases (unhandled first or vice versa), behaviour, body weight and, for corresponding analyses, comb or wattle size were covariates, retained in the model to control for other sources of variation than handling on skin temperature. Time of day and the random effect group also control for any temporal variation in hormone levels.

## 3 Results

Wattle and comb temperature revealed stressor intensity (Fig. 1B, C, Table 1). Firstly, whilst little temporal change was evident in the unhandled trial, skin temperature immediately dropped from baseline following both holds, but was significantly lower in side-pinned than cradled hens (model in Table 1 compared to same model with cradled and side-pinned hold types collapsed together, wattle LRT X^2^_1_ = 40.00, p < 0.0001; comb LRT X^2^_1_ = 111.8, p < 0.0001). The drop from baseline levels in wattle and comb temperature was 0.7 °C and 0.5 °C respectively in cradled hens, compared to 1.3 °C and 2.2 °C respectively in side-pinned hens. Secondly, side-pinning, only, also induced a post-stressor increase in temperature above baseline (wattle: 10 min post-handling, comb: 17 min). This may indicate greater dissipation of core heat [12], [13], and provides a second time point for the quantification of stress. Wattle temperature was lowest immediately following release whilst comb temperature continued to drop for 5 min, before increasing. As such, there was an overall linear relationship between seconds into the trial and wattle temperature but a quadratic relationship between seconds into the trial and comb temperature (wattle: removal of (seconds)^2^ × trial LRT X^2^_2_ = 0.51, p = 78, removal of seconds × trial LRT X^2^_2_ = 110.2, p < 0.0001; comb: removal of (seconds)^2^ × trial LRT X^2^_2_ = 20.91, p < 0.0001). Whilst eye temperature also dropped around 0.4 °C with handling (Table 1), the extent and temporal pattern did not differ between handling types (removal of (seconds)^2^ x trial LRT X^2^_2_ = 2.06, p = 0.36, removal of seconds x trial LRT X^2^_2_ = 1.99, p = 0.37, removal of trial from model LRT X^2^_2_ = 245.2, p < 0.0001, collapsing together handling trials: LRT X^2^_1_ = 3.13, p = 0.08), which suggests that it can identify but should not be used to quantify acute stress in hens. Facial temperature did not significantly differ between trials, in interaction with seconds from release or as a main effect (removal of (seconds)^2^ × trial LRT X^2^_2_ = 5.13, p = 0.08, removal of seconds × trial LRT X^2^_2_ = 4.84, p = 0.09, removal of trial from model LRT X^2^_2_ = 1.59, p = 0.45). All facial regions were cooler when hens were active than inactive (Table 1). As expected, temporal patterns were strongest in hens with relatively high baseline temperature (Table 1). Analyses also control for significant effects of camera distance and body position and non-significant effects of variation in comb or wattle size and body weight on temperature by retaining these in the model (Table 1).

## 4 Discussion

The extent of skin cooling and subsequent warming reflected acute stressor intensity when observed in the comb and wattle, but not in the eye or face. In chickens, the comb and wattle have higher densities of arteriovenous anastomoses (AVAs), the arteriole–venule connections that bypass capillary beds, than in the face [16]. Cutaneous AVAs have an important role in thermoregulation: whilst capillary flow is more closely linked to local skin temperature, blood flow through AVAs increases in proportion to core body temperature, allowing core heat dissipation under heat stress or retention under cold stress [19], [20]. That stress-induced, graded temperature changes were observed in these specific skin regions is consistent with a link between skin surface patterns and SIH in the core. In mammals, too, stress-induced vasoconstriction is most pronounced in extremities relatively rich in AVAs, such as rat tails [3], rabbit ears [21] and human fingers [22], providing targets for the translation of this method to other species.

Eye temperature, in contrast, decreased with handling, consistent with previous research [10], but did not exhibit proportionality with stressor intensity. Edgar et al. [23] similarly found that in chicken chicks, which do not have a comb or wattle, the magnitude of an eye temperature drop when exposed to an acute stressor (a puff of air) was not increased by simultaneous application of a social stressor (absence of the mother). As such, eye temperature may identify but not be useful for quantifying acute stress in chickens. In other species where much of the body is insulated by feathers or fur, bare-skinned, AVA-rich regions may not be available, though, and the thermal window of the eye may play a more significant role in thermoregulation [24]. As such, this study supports skin temperature change as a potential marker of acute stress intensity, but age- and species-specific validations of the optimal skin regions are required.

We make three further practical recommendations for the use of skin temperature change as a measure of acute stress in other species. First, the initial skin temperature drop can be rapid, so to capture the minimum, measurements should be taken at intervals significantly shorter than the anticipated speed of the response, and interpreted in relation to the exact timing of measurement. This is likely to be species-specific. For example, wattle and comb temperature reached minimum values within one and five minutes of handling, respectively, captured with a 10s sampling interval, whilst a restraint stressor (trapping in a feeding box) caused the skin around the eye to drop by around 2 °C within 10 s in blue tits (*Cyanistes caeruleus*) [9]. Second, given the presence, still, of skin temperature differences between mild and more severely stressed hens at 20 min post-handling, it is imperative that other sources of stress are minimized or carefully recorded prior to stress manipulation. Sequential testing without sufficient recovery periods may cause carryover between trials [25]. Third, individual-level studies require individual baseline measurements. Skin temperature varied consistently amongst unstressed hens, and pre-stressor skin temperature influenced the magnitude of the temperature response. Given the rapidity of the response to handling, baseline skin temperature should be measured in situ prior to any data collection requiring capture for measurement [11].

That facial regions were cooler when hens were active than inactive suggests physical movement, expected to generate heat, did not determine skin temperature. In studies on mammals, activity under stress has little impact on SIH in the core [5], [26], nor does muscular movement associated with stress-related facial expressions alter facial skin temperature [27]. So, skin temperature and behaviour may provide independent and complementary information on stress state.

For skin temperature measurement, IRT is a non-invasive alternative to conductive devices that are attached directly to the skin. IRT therefore allows continuous measurement of skin temperature without the confounding effects of carrying a device on stress levels, behaviour, or skin microclimate [10]. With regard to acute stress, the non-invasive nature of IRT overcomes a major limitation of both hormonal and core temperature techniques, by allowing time-series data collection within individuals without repeated handling, blood sampling and/or probe insertion that alter downstream stress levels [6], [7]. That two time points, the immediate skin cooling and subsequent warming, could distinguish mild from more severe acute stress in this study already demonstrates the value of continuous, noninvasive measurement. Describing the whole course of the stress response opens new avenues into individual-level research, where variation in the duration and magnitude of the hormonal stress response varies amongst individuals and correlates with ecologically important differences in their behaviour and physiology [17].

Core temperature is linearly related to stress level in mice [7]. The scope for SIH to detect the anxiogenic effect of pharmaceuticals is limited by ceiling effects in core temperature [4], particularly when the stress-inducing effects of repeated handling or probe insertion must be incorporated [28]. Whilst skin temperature can be measured non-invasively [10] and is inherently more variable and dynamic than core temperature [15], [22], whether it could be a viable alternative depends on whether it is similarly, linearly related to stressor intensity. A linear relationship between stressor intensity and skin temperature would be expected, as part of the same acute, physiological stress response that includes linear corticosterone and core temperature increases [7], [17]. We show here that the change in skin temperature to an acute stressor is not simply an ‘on-off phenomenon’, but that two different stressor intensities resulted in different skin temperatures. However, testing the linearity of the relationship, and identifying the upper limits of the response, will requires a continuous or larger range of stressor intensities than the two applied in this study, and further cross-validation of skin temperature with core temperature or corticosterone levels collected from the same individual. Moreover, the linearity of the response would need to be assessed in a broader sample of the population than here, as one study on humans identified sex-specific patterns in skin temperature [22], and others age-related declines in vasoconstriction [29]. Finally, the response would need to be examined under different ambient temperatures, as the magnitude of SIH [30] and blood flow through AVAs [31] varies with ambient temperature. Indoor farmed and laboratory animals are maintained under a relatively constant and narrow temperature range, and chickens kept between 18 °C and 23 °C (here and [12], [13]) show similar skin temperature responses to handling. For outdoor domestic or wild animals, additional steps will be required to carefully measure and validate the skin temperature response, and indeed evaluate the independence of activity and skin temperature, under different ambient conditions [9], [32]. However, this study provides a vital, first step toward applying this method to stress assessment.

## 5 Conclusions

We provide a proof of concept: that skin temperature can indicate acute stressor intensity, and also demonstrate congruence between hormonal, behavioural and skin thermal patterns. Stress-induced temperature changes appear to reflect a cognitive process, with one bovine study reporting no effect of artificially increasing cortisol or epinephrine, the hormones associated with an acute stress response, on eye or core temperature when administered in isolation from any experience of a stressor [33]. Indeed, acute psychological stressors, alone, can elicit SIH [2]. The same hormones are elevated under chronic stress [34], and ongoing psychological challenges are known to increase core temperature in rats [34], [35]. Whether chronic stress would also manifest in long term lowered skin temperature, as observed in fingertip temperature with depression in humans [36], or instead long term raised skin temperature, to dissipate heat from elevated core temperatures [34], [35], is unknown, and would be a valuable line of enquiry in animal welfare assessment. Beyond stress however, increased mental workload [37], pain [38] and even positive experiences [25], [39] can trigger an acute drop in skin temperature. Whilst this ubiquity stems from shared, underlying hormonal changes in different emotional states, such that the skin temperature changes here may reflect arousal rather than stress specifically [1], in some species, variation in the responsiveness of different skin regions to positive versus negative stimuli suggests that comparison of temperature changes across skin regions may also reveal emotional valence [11]. Moreover, pronounced changes in the distribution of heat around the body occur during thermoregulation [14], [15]. Consideration of the skin region selected and the emotional and physical context will clearly be important to understanding patterns of skin temperature change. With stress central to health, welfare and physiological function, though, as a non-invasive and proportional marker of stress, skin temperature changes are likely to form the basis of novel assays in animal welfare [40], medical diagnostics [31] and ecology [10].

## Figures and Tables

**Table 1:** *n* = 5065–5354 measurements of the face, eye, wattle and comb of 57 individuals. Models presented are the reduced linear mixed models after removal of non-significant interactions of trial × seconds (from trapping in or release into the film area) and main effects of trial. Standard deviations (σ) of the random effects group and ID nested in group, and residual variation, are reported. In the factor ‘trial’, holds are compared to the unhandled treatment.

Variable	Face temperature				Eye temperature				Wattle temperature				Comb temperature			
Value	S.E.	*T*	*p*	Value	S.E.	*t*	*p*	Value	S.E.	*t*	*p*	Value	S.E.	*t*	*p*
σ group	0.09				0.12				0.16				0.00			
σ ID nested in group	0.12				0.22				0.43				1.50			
σ residual	0.83				0.84				1.54				2.58			
Intercept	13.99	1.56	8.99	< 0.0001	11.58	1.83	6.32	< 0.0001	6.86	2.34	2.93	0.003	10.89	4.13	2.64	0.008
Seconds	0.00	0.00	0.73	0.46	0.00	0.00	0.37	0.71	0.00	0.00	6.28	< 0.0001	0.00	0.00	1.00	0.32
(Seconds)^2^	0.00	0.00	− 1.04	0.30	0.00	0.00	− 1.33	0.19	0.00	0.00	− 5.59	< 0.0001	0.00	0.00	− 1.27	0.20
Baseline temperature	− 0.39	0.04	− 9.59	< 0.0001	− 0.38	0.06	− 6.41	< 0.0001	− 0.22	0.05	− 3.97	0.0003	− 0.31	0.07	− 4.66	< 0.0001
Time of day	0.00	0.00	2.08	0.038	0.00	0.00	0.20	0.84	0.00	0.00	1.29	0.20	− 0.00	0.00	− 2.56	0.011
Head position — up	− 0.07	0.06	− 1.26	0.21	− 0.31	0.06	− 5.25	< 0.0001	0.26	0.10	2.44	0.015	0.47	0.18	2.67	0.008
Face angle – flat	− 0.05	0.07	− 0.78	0.43	0.21	0.07	2.90	0.004	− 0.11	0.13	− 0.85	0.39	0.21	0.21	0.97	0.33
Face angle — up	− 0.15	0.08	− 1.79	0.07	0.37	0.09	4.13	< 0.0001	− 0.35	0.16	− 2.26	0.029	0.65	0.26	2.46	0.014
Head tilt — side on	0.11	0.05	2.07	0.039	− 0.19	0.06	− 3.39	0.001	− 0.34	0.10	− 3.33	0.0009	− 1.41	0.17	− 8.22	< 0.0001
Head tilt — away	− 0.07	0.09	− 0.83	0.41	− 0.21	0.09	− 2.23	0.026	− 0.97	0.16	− 5.93	< 0.0001	− 1.41	0.27	− 5.16	< 0.0001
Active — yes	− 0.25	0.03	− 7.57	< 0.0001	− 0.42	0.04	− 11.60	< 0.0001	− 0.43	0.06	− 6.84	< 0.0001	− 0.83	0.11	− 7.82	< 0.0001
Distance — front	1.08	0.03	31.86	< 0.0001	0.45	0.04	12.62	< 0.0001	0.76	0.06	11.91	< 0.0001	1.39	0.11	12.96	< 0.0001
Distance — back	0.42	0.03	12.36	< 0.0001	0.03	0.04	0.73	0.47	0.34	0.07	5.14	< 0.0001	0.45	0.11	4.03	0.0001
Side — right	0.06	0.02	2.49	0.013	0.16	0.02	6.31	< 0.0001	− 0.03	0.04	− 0.55	0.58	0.07	0.07	0.92	0.36
Weight	− 0.02	0.15	− 0.12	0.91	0.22	0.24	0.92	0.36	0.11	0.49	0.22	0.83	0.47	1.61	0.29	0.77
Region size	n/a	n/a	n/a	n/a	n/a	n/a	n/a	n/a	0.06	0.07	0.91	0.37	0.01	0.08	0.17	0.87
Trial — cradled	–	–	–	–	− 0.47	0.03	− 13.49	< 0.0001	− 0.67	0.09	− 7.22	< 0.0001	− 0.77	0.22	− 3.52	0.0004
Trial — side	–	–	–	–	− 0.39	0.04	− 10.49	< 0.0001	− 1.25	0.10	− 12.63	< 0.0001	− 1.94	0.23	− 8.47	< 0.0001
Seconds × trial — cradled	–	–	–	–	–	–	–	–	0.00	0.00	3.55	0.0004	0.00	0.00	0.94	0.35
Seconds × trial — side-pinned	–	–	–	–	–	–	–	–	0.00	0.00	10.53	< 0.0001	− 0.00	0.00	− 1.82	0.07
(Seconds)^2^ × trial — cradled	–	–	–	–	–	–	–	–	–	–	–	–	− 0.00	0.00	− 1.00	0.32
(Seconds)^2^ × trial — side-pinned	–	–	–	–	–	–	–	–	–	–	–	–	0.00	0.00	3.89	0.0001

## References

[1] Buwalda B., Scholte J., de Boer S.F., Coppens C.M., Koolhaas J.M. (2012 Feb). The acute glucocorticoid stress response does not differentiate between rewarding and aversive social stimuli in rats. Horm. Behav..

[2] Oka T., Oka K., Hori T. (2001). Mechanisms and mediators of psychological stress-induced rise in core temperature. Psychosom. Med..

[3] Marks A., Vianna D.M.L., Carrive P. (2009 Feb 4). Nonshivering thermogenesis without interscapular brown adipose tissue involvement during conditioned fear in the rat. AJP Regul. Integr. Comp. Physiol..

[4] Bouwknecht J.A., Olivier B., Paylor R.E. (2007 Jan). The stress-induced hyperthermia paradigm as a physiological animal model for anxiety: a review of pharmacological and genetic studies in the mouse. Neurosci. Biobehav. Rev..

[5] Bakken M., Moe R.O., Smith A.J., Selle G.-M.E. (1999). Effects of environmental stressors on deep body temperature and activity levels in silver fox vixens (*Vulpes vulpes*). Appl. Anim. Behav. Sci..

[6] Lagadic H., Faure J.M., Mills A.D., Williams J.B. (1990 Dec). Effects of blood sampling on plasma concentrations of corticosterone and glucose in laying hens caged in groups. Br. Poult. Sci..

[7] Veening J.G., Bouwknecht J.A., Joosten H.J.J., Dederen P.J., Zethof T.J.J., Groenink L. (2004 Jul). Stress-induced hyperthermia in the mouse: c-fos expression, corticosterone and temperature changes. Prog. Neuro-Psychopharmacol. Biol. Psychiatry.

[8] Korte S.M., Beuving G., Ruesink W., Blokhuis H.J. (1997). Plasma catecholamine and corticosterone levels during manual restraint in chicks from a high and low feather pecking line of laying hens. Physiol. Behav..

[9] Jerem P, Herborn KA, McCafferty DJ, McKeegan D, Nager R. Thermal imaging to study stress non-invasively in unrestrained birds. J. Vis. Exp.:(in press). 10.3791/53184 PMC4692699 26575985

[10] McCafferty D.J. (2013). Applications of thermal imaging in avian science. Ibis.

[11] Ioannou S., Gallese V., Merla A. (2014 Oct). Thermal infrared imaging in psychophysiology: potentialities and limits: thermal infrared imaging in psychophysiology. Psychophysiology.

[12] Edgar J.L., Nicol C.J., Pugh C.A., Paul E.S. (2013 Jul). Surface temperature changes in response to handling in domestic chickens. Physiol. Behav..

[13] Cabanac M., Aizawa S. (2000). Fever and tachycardia in a bird (*Gallus domesticus*) after simple handling. Physiol. Behav..

[14] Edens F.W., Siegel H.S. (1975). Adrenal responses in high and low ACTH response lines of chickens during acute heat stress. Gen. Comp. Endocrinol..

[15] Giloh M, Shinder D, Yahav S. Skin surface temperature of broiler chickens is correlated to body core temperature and is indicative of their thermoregulatory status. Poult. Sci. 2012 Jan 1;91(1):175–88. 10.3382/ps.2011-01497 22184442

[16] Kannan G., Mench J.A. (1996 Mar). Influence of different handling methods and crating periods on plasma corticosterone concentrations in broilers. Br. Poult. Sci..

[17] Cockrem J.F. (2007 Dec). Stress, corticosterone responses and avian personalities. J. Ornithol..

[18] Mollenhorst H., Rodenburg T.B., Bokkers E.A.M., Koene P., de Boer I.J.M. (2005 Mar). On-farm assessment of laying hen welfare: a comparison of one environment-based and two animal-based methods. Appl. Anim. Behav. Sci..

[19] Hales JRS, Fawcett AA, Bennett J. T. Differential influences of CNS and superficial body temperatures on the partition of cutaneous blood flow between capillaries and arteriovenous anastomoses (AVA's). Pflugers Arch. 1975;361:105–6. 10.1007/BF00587350 1239737

[20] Wolfenson D., Frei Y.F., Snapir N., Berman A. (1981). Heat stress effects on capillary blood flow and its redistribution in the laying hen. Pflugers Arch..

[21] Yu Y.H., Blessing W.W. (1999). Amygdala co-ordinates sudden falls in ear pinna blood flow in response to unconditioned salient stimuli in conscious rabbits. Neuroscience.

[22] Vinkers C.H., Penning R., Hellhammer J., Verster J.C., Klaessens J.H.G.M., Olivier B. (2013 Sep). The effect of stress on core and peripheral body temperature in humans. Stress.

[23] Edgar J., Held S., Paul E., Pettersson I., I'Anson Price R., Nicol C. (2015 Jul). Social buffering in a bird. Anim. Behav..

[24] Bech C., Midtgård U. (1981 Jun). Brain temperature and the *rete mirabile ophthalmicum* in the zebra finch (*Poephila guttata*). J. Comp. Physiol..

[25] Ioannou S., Chotard H., Davila-Ross M. (2015 Jun). No strings attached: physiological monitoring of rhesus monkeys (*Macaca mulatta*) with thermal imaging. Front. Behav. Neurosci..

[26] Meyer L.C., Fick L., Matthee A., Mitchell D., Fuller A. (2008). Hyperthermia in captured impala (*Aepyceros melampus*): a fright not flight response. J. Wildl. Dis..

[27] Nakayama K., Goto S., Kuraoka K., Nakamura K. (2005 Apr). Decrease in nasal temperature of rhesus monkeys (*Macaca mulatta*) in negative emotional state. Physiol. Behav..

[28] Van Der Heyden J.A., Zethof T.J., Olivier B. (1997). Stress-induced hyperthermia in singly housed mice. Physiol. Behav..

[29] DeGroot D.W., Kenney W.L. (2006 Sep 7). Impaired defense of core temperature in aged humans during mild cold stress. AJP Regul. Integr. Comp. Physiol..

[30] Briese E. (1992). Cold increases and warmth diminishes stress-induced rise of colonic temperature in rats. Physiol. Behav..

[31] Krogstad A.-L., Elam M., Karlsson T., Wallin B.G. (1993). Arteriovenous anastomoses and the thermoregulatory shift between cutaneous vasoconstrictor and vasodilator reflexes. J. Auton. Nerv. Syst..

[32] Fernández-Cuevas I., Bouzas Marins J.C., Arnáiz Lastras J., Gómez Carmona P.M., Piñonosa Cano S., García-Concepción M.Á. (2015 Jul). Classification of factors influencing the use of infrared thermography in humans: a review. Infrared Phys. Technol..

[33] Stewart M., Webster J.R., Verkerk G.A., Schaefer A.L., Colyn J.J., Stafford K.J. (2007 Oct). Non-invasive measurement of stress in dairy cows using infrared thermography. Physiol. Behav..

[34] Keeney AJ, Hogg S, Marsden CA. Alterations in core body temperature, locomotor activity, and corticosterone following acute and repeated social defeat of male NMRI mice. Physiol. Behav. 2001;74(1):177–84. 10.1016/s0031-9384(01)00541-8 11564466

[35] Endo Y., Shiraki K. (2000). Behavior and body temperature in rats following chronic foot shock or psychological stress exposure. Physiol. Behav..

[36] Lin H.-P., Lin H.-Y., Lin W.-L., Huang A.C.-W. (2011 Oct). Effects of stress, depression, and their interaction on heart rate, skin conductance, finger temperature, and respiratory rate: sympathetic-parasympathetic hypothesis of stress and depression. J. Clin. Psychol..

[37] Genno H., Ishikawa K., Kanbara O., Kikumoto M., Fujiwara Y., Suzuki R. (1997). Using facial skin temperature to objectively evaluate sensations. Int. J. Ind. Ergon..

[38] Stewart M., Stafford K.J., Dowling S.K., Schaefer A.L., Webster J.R. (2008 Mar). Eye temperature and heart rate variability of calves disbudded with or without local anaesthetic. Physiol. Behav..

[39] Moe R.O., Nordgreen J., Janczak A.M., Bakken M., Spruijt B.M., Jensen P. (2014 Jun). Anticipatory and foraging behaviors in response to palatable food reward in chickens: effects of dopamine D2 receptor blockade and domestication. Physiol. Behav..

[40] McGreevy P., Warren-Smith A., Guisard Y. (2012 May). The effect of double bridles and jaw-clamping crank nosebands on temperature of eyes and facial skin of horses. J. Vet. Behav. Clin. Appl. Res..

